# Advancing outbreak detection: Hybridizing machine learning with wavelets for weekly dengue case forecasting

**DOI:** 10.1371/journal.pntd.0014444

**Published:** 2026-07-09

**Authors:** Angelica Anne Eligado, Takanori Hasegawa, Yuta Hattori, Keiko Nakamura

**Affiliations:** 1 Department of Global Health Entrepreneurship, Institute of Science Tokyo, Tokyo, Japan; 2 Department of Epidemiology and Biostatistics, College of Public Health, University of the Philippines-Manila, Manila, Philippines; 3 Department of Integrated Analytics, Medical and Dental Data Science Center, Institute of Science Tokyo, Tokyo, Japan; 4 Department of Preventive Medicine, Nagoya University Graduate School of Medicine, Nagoya, Japan; Christian Medical College Vellore, INDIA

## Abstract

**Background:**

Traditional surveillance systems often struggle with the volatility of weekly case data, limiting timely prevention and control efforts. In the Philippines, the standard method for setting outbreak thresholds relies on historical moving averages that are highly affected by extreme values and slow to reflect recent epidemiological shifts. This study assessed the performance of hybrid discrete wavelet transform (DWT)-seasonal autoregressive moving average (SARMA) and DWT-SARMA-long short-term memory (LSTM) models in forecasting weekly case counts and explored their potential in defining dynamic alarm and epidemic thresholds in Quezon City, Philippines.

**Methodology:**

An ecologic time-trend study was conducted using weekly dengue case counts from 2012 to 2022. The data was decomposed using DWT, and SARMA was applied to the resulting approximate and detail coefficients. The DWT-SARMA model was enhanced by applying an LSTM to the SARMA residuals. The DWT-SARMA-LSTM model demonstrated superior performance, achieving a Mean Absolute Percentage Error (MAPE) of 12.4%, and successfully captured the case peaks and troughs. In contrast, the DWT-SARMA model produced a MAPE of 25.8%. Model-derived thresholds were more adaptive and context-sensitive than the traditional 3-year moving mean threshold, which was skewed by pre-pandemic data.

**Conclusion:**

The hybrid DWT-SARMA-LSTM model is an accurate and robust approach for forecasting weekly dengue cases. It provides a more responsive basis for an early warning system than traditional thresholding methods, and has practical value for outbreak detection and resource planning in dynamic public health environments, particularly in resource-limited settings where timely and accurate data are critical.

## Author summary

Dengue fever is a mosquito-borne disease that affects millions of people, especially in tropical and subtropical regions. This study focused on improving forecasting future dengue outbreaks in Quezon City, Philippines, using weekly case data collected over more than a decade. The goal was to develop a forecasting method that captures both long-term patterns and sudden changes in dengue cases. To achieve this, we combined two approaches: one approach that identifies regular trends in the data with another that learns from unpredictable changes. We also used a technique that breaks down data into simpler parts, enabling us to understand and model the disease more effectively. By testing different combinations of methods, we identified a setup that produced accurate forecasts up to 17 weeks ahead. These forecasts can support local health authorities in preparing for potential outbreaks by setting early warning thresholds. Our approach offers a practical tool for improving disease surveillance and response in areas where dengue is a recurring threat and may contribute to more timely and targeted public health actions that reduce the impact of dengue on communities.

## Introduction

Dengue is a major public health concern. Globally, the number of reported cases increased by more than ten-fold from 505 thousand cases in 2000 to 5.2 million in 2019 [1]. Although Dengvaxia and Qdenga, are registered for the dengue prevention, Dengvaxia requires pre-vaccination screening and is restricted to individuals who tested positive for prior infection, limiting use [2]. Qdenga does not provide complete protection against dengue [2]. Therefore, the World Health Organization emphasizes the importance of a comprehensive vector control program and effective surveillance mitigate dengue [3].

Public health surveillance involves the systematic collection, analysis, and interpretation of health-related data to inform public health actions, prevent and control diseases, and identify events that are of public health concern [4,5]. Integrated surveillance and outbreak preparedness are among the five major components in reducing morbidity, mortality, and burden of dengue [3]. Information obtained from surveillance enables program planners to understand the distribution of cases and their entomological correlates, enabling targeted prevention and response strategies. However, prevention and control measures are frequently delayed and initiated during the late emergency phase when the number of cases is already declining [6] An algorithm for predicting the spread of disease and defining the alert and outbreak thresholds is needed for early warning and timely prevention and control measures [7].

Definitions of outbreaks vary across countries and areas [6,8]. Epidemiologic criteria, the most common method for defining an outbreak, involves distinguishing between the expected number of cases and excessive case counts [6]. The epidemiologic criteria comprise two key components: (i) the endemic channel, which reflects historical trends of expected cases, and (ii) the criteria that determine the threshold of variation above the endemic channel that is classified as an outbreak [9] Traditional methods for calculating the endemic channel, such as moving average, recent mean, cumulative mean, and historical limits, are based on historical means [10]. These methods have an underlying assumption of independence of observations, an assumption that is often violated in disease surveillance due to serial dependence inherent in regularly collected [11]. On the other hand, the threshold of variation is typically set to two standard deviations above the endemic channel [9,12].

Evidence indicates that methods relying on historical means plus two standard deviations for defining the endemic threshold have led to suboptimal response plans, prompting jurisdictions to modify the epidemiologic criteria for local use [6,8]. This lack of standardization hampers global efforts to establish uniform criteria for what constitutes an outbreak and may impede the development of consistent, evidence-based strategies [9]. Furthermore, mean-based methods for computing the endemic channel vary widely in their ability to detect an outbreak, as demonstrated by Brady et al. (2014) [13]. Even when applied to the same dataset, significant differences in the number and proportion of identified outbreaks were observed, with greater variation in specific regions [8]. The unreliability and heterogeneity of performance of common methods for detecting an outbreak can be attributed to the complexity of disease transmission dynamics. These dynamics are inherently complicated by the extensive genetic diversity across four distinct dengue serotypes and are additionally modulated by diverse environmental factors that affect transmission patterns [14–17].

The variability in performance of outbreak detection methods and the complex transmission patterns, which is driven by the interaction among vector, pathogen, human and environment, have led to the development of models incorporating climate data [18–21] Among these models, the Early Warning System and Response System (EWARS) for dengue outbreaks is widely recognized [7,22,23]. The EWARS assists countries in establishing early warning mechanisms for efficient and cost-effective local responses by applying outbreak and alarm indicators to construct forecasting models capable of anticipating dengue outbreaks at the district level [22]. Although EWARS is currently implemented in 17 dengue endemic countries [24], most countries have not adopted it and challenges in collecting near-real-time climate data persist, particularly in middle-income countries. Thus, a method that detects meaningful surges in temporal data remains essential for timely outbreak detection and response.

The discrete wavelet transform (DWT) is a technique for decomposing time-series data into multiple components using wavelet functions, which separate the data into distinct frequencies at various scales through the application of high-pass and low-pass filters [25–26]. This process produces approximate (cA) and detail (cD) coefficients, down sampling the original data by a factor of 2 at each decomposition level. The cA captures the broad, low-frequency features of the data, such as seasonal patterns or long-term shifts, while cD highlights high-frequency components, including short-term fluctuations, and abrupt changes that may be obscured by dominant trends and seasonality [25–27]. DWT enhances out-of-sample forecasting accuracy compared to traditional methods, such as the autoregressive integrated moving average (ARIMA) model, because of its ability to segregate trends, seasonality, and noise from the data [28]. While DWT is mathematically invertible and allows the reconstruction of the original data from the resulting coefficients, this property is more relevant in a signal processing context. In forecasting applications, the goal is not reconstruction but the extraction of informative features and noise reduction, thereby improving model performance.

Due to the ability of DWT to reduce noise and preserve the components of data containing valuable information [29–30], it has become a tool for a wide range of domains. Its applications include dimensionality reduction, time-series classification, clustering, trend and anomaly detection, and prediction modelling [26–27]. In the last 20 years, its use in health has gained momentum, with studies applying DWT for outbreak detection and forecasting of various infectious diseases, such as gastrointestinal complaints [31], influenza [32], and pertussis [33] and even bioterrorism [11,34]. Hybrid models combining DWT with seasonal autoregressive moving average (SARMA) or ARIMA yielded satisfactory results in forecasting the number of COVID-19 cases [35–37]. However, research applying DWT for detecting outbreaks and forecasting dengue cases remains limited, demonstrating a need for further investigation.

Univariate long short-term memory (LSTM) networks are a promising deep learning method for disease prediction when prior knowledge of factors affecting disease transmission is limited [38]. LSTM networks model dependencies between future values and past time steps and can learn oscillating behaviors and seasonality in time series [39]. These capabilities make LSTM networks suitable for modeling seasonal diseases [15]. Unlike ARIMA and SARMA, which require parameter estimation and can be time-consuming for large datasets, LSTM networks are non-parametric and do not have this limitation [38]. LSTM has been widely used in healthcare forecasting, including forecasting the number of COVID-19 cases [40], influenza [41], and dengue cases [42,43]. Necesito et al. (2021) applied a hybrid DWT-LSTM model to forecast dengue cases in the Philippines [15] with high accuracy, although forecasts were generated on a monthly scale. With the exceptions of a few [44–47], majority of forecasting models in the literature predicted the monthly number or incidence of dengue cases. While monthly forecasts of dengue cases are invaluable for dengue prevention and control, predictions at a smaller time scale, notably weekly, are necessary for early outbreak detection and response [44].

Given encouraging results from the application of DWT, LSTM, and hybrid approaches for forecasting disease spread, existing limitations in detecting signals of dengue outbreaks, this study applies these methods to weekly dengue surveillance. The objective is to evaluate the performance of DWT-SARMA and DWT-SARMA-LSTM models in forecasting the weekly number of dengue cases in Quezon City, Philippines. A secondary objective is to examine the potential of univariate models to define alert and epidemic thresholds for dengue through DWT method.

## Methods

### Study population

The study population is the residents of Quezon City, Philippines, the largest city in the National Capital Region (NCR). Based on the 2020 Census, the population is 2,960,048, with a population density of 17,239 persons per square kilometer, making it one of the most densely populated urban areas in the country [48,49]. Administratively, it comprises 142 villages, locally referred to as barangays, which can be further grouped into six legislative districts [49].

Dengue is one of the public health concerns in the city, as Quezon City is among the areas with a high incidence and mortality rate in the Philippines [50–52]. Given the city’s densely populated urban environment, coupled with high dengue incidence and mortality rates, Quezon City presents a critical setting for epidemiological research and public health interventions.

### Data acquisition and preprocessing

Anonymized data of routinely collected dengue cases from January 2012 to December 2022 were provided by the Quezon City Epidemiology and Surveillance Unit (QCESU) as a Microsoft Excel file. Under the Philippine Integrated Disease Surveillance and Response system, all health service delivery units in the city are required to submit weekly reports of all dengue cases to Epidemiology and Surveillance Units. The unit of analysis is dengue cases, which are assigned the date of admission, the name of the disease reporting unit, the barangay of residence, and the case classification (suspected, probable, and confirmed). All cases, regardless of case classification, were included in the analysis. The data is proprietary and not publicly available.

**Fig 1:**
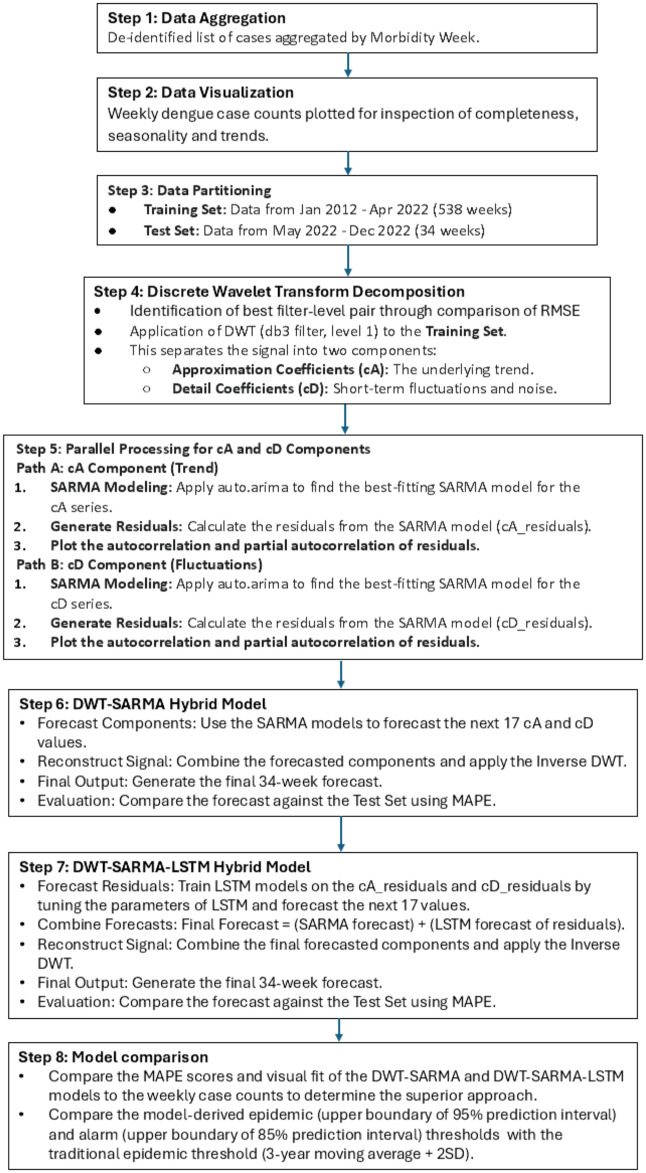
Steps in data preprocessing and computation of forecasts and thresholds from the DWT-SARMA and DWT-SARMA-LSTM models.

The process of preprocessing and analysis is summarized in Fig 1. The original data provided a daily number of cases based on the date of admission. The authors further aggregated them into weekly case counts and checked the completeness. This aggregation was performed based on the Morbidity Week (MW), which is the standard reporting unit of time used by the PIDSR for epidemiological tracking. No missing data point at the week level was found. The aggregated dataset was partitioned into training and test sets. The training data used for forecasting consists of 538 weekly data points from January 2012 (MW 1) to April 2022 (MW 18). The remaining data points for the subsequent 34 weeks, from May to December 2022, were used for testing the out-of-sample forecast accuracy. No other transformations were applied to the data before decomposition using DWT. We developed and compared two hybrid models: a DWT-SARMA model and a DWT-SARMA-LSTM model. The entire workflow was implemented in Python using PyWavelets [53] statsmodel [54], Keras [55], and pmdarima [56] libraries.

### Statistical analysis

An ecological time trend study design was employed in developing the models for forecasting of weekly cases. First, we performed a Discrete Wavelet Transform decomposition on the test data to obtain cA and cD components using Pywavelets library [53]. This process corresponds to step 4 in Fig 1. The cA components capture the low-frequency structure of the data, representing the underlying trends. In contrast, the cD components retain high-frequency information, representing short-term fluctuations, transient dynamics, and noise. To identify the optimal wavelet filter and decomposition level, we automated the reconstruction of the decomposed time series across multiple filter-level combinations and selected the pair that yielded the lowest Root Mean Square Error (RMSE). Wavelet filters are mathematical tools that breakdown the time series into its low and high-frequency components while the decomposition level refers to how many times the wavelet filter was applied, with each level progressively simplifying the time series. Daubechies 3, a family of wavelets, at level 1, was determined to have the lowest RMSE value and was selected as the parameter to be used onwards. The Daubechies (Db) family of wavelets was chosen for its efficiency in signal analysis, with the Db3 filter providing a good balance between smoothness and compactness.

Next, we used the auto.arima function from the pmdarima library [56]in Python to select the best-fitting SARMA models for cA and cD independently. This process corresponds to step 5 in Fig 1. The function automatically iterates through all possible models and returns the one with the best fit according to the Akaike Information Criterion (AIC). As described in step 6, we then computed horizons (*h*) of 17 data points for both cA and cD components. The number of horizons, 17, was chosen because the test set consists of 34 MWs (MW 19 *–* 52), and the DWT process at level 1 downsamples the frequency of the data points by a factor of two, resulting in a 17-MW forecast horizon for each component.

We applied an LSTM neural network to the SARMA residuals to capture any nonlinear patterns not modeled by the SARMA. Residuals are the differences between the observed weekly case counts and the predictions of the SARMA model. This process, described as step 7 in Fig 1, involved three steps. First, we determined the optimal number of look-back points, which is the number of previous time points that the LSTM should consider when making predictions, by examining the autocorrelation function and partial autocorrelation function of the residuals following the methodology by Khandelwal et. al. (2015) [57]. Next, the residuals were rescaled to a range of [0,1] using min-max normalization to ensure model stability. Each residual data point was converted into values ranging from 0 to 1 by subtracting each data point from the minimum value in the time series and then dividing it by the difference between the maximum and minimum values [58]. The formula for rescaling is given by:


$$\begin{array}{c} x_{scaled}=\frac{x_{i}-x_{min}}{x_{max}-x_{min}}, \end{array}$$

where *x*_*scaled*_ is the normalized value, *x*_*i*_ is the residual at data point *i*, and *x*_*max*_ and *x*_*min*_ are the maximum and minimum values in the decomposed time series data. Finally, a two-layer LSTM model was constructed, consisting of a sequential data processing layer and a dense forecasting layer. To prevent overfitting, we implemented early stopping with a patience parameter of two, using the mean squared error as the loss metric. The optimizer is set to Adaptive Moment estimation (Adam) [59] with a default learning rate of 0.001 and a batch size of four. As a result, hyperparameters were tuned empirically through a series of experiments in which a total of 500 models were evaluated. The optimal number of epochs was determined by running 10 iterations for each epoch configuration, ranging from 10 to 300 in increments of 10. The distribution of the error scores computed from the iterations was evaluated, and the number of epochs with the lowest mean error score and narrowest interval was selected. A similar procedure tuned the number of neurons, ranging from one to 20, and 10 iterations were performed for each configuration. The neuron count with the lowest mean error and the smallest variability was selected. The final configuration consisted of 90 training epochs, with three neurons for the cA residuals and one neuron for the cD residuals.

The final forecast was constructed by coupling the different model components. This process corresponds to step 8 in Fig 1. For the DWT-SARMA-LSTM model, the forecasted residuals of the cA and cD components from the LSTM models were added to the forecasted values of cA and cD components from the SARMA models. The sum was then appended to the training cA and cD data. For both the DWT-SARMA and DWT-SARMA-LSTM models, the final forecast data from the combined components were reconstructed using an inverse DWT. Point estimates and their 95.5% prediction interval estimates, which correspond to +2-standard deviation of the point estimates, were generated. The accuracy of the out-of-sample forecasts was evaluated using the Mean Absolute Percentage Error (MAPE), interpreted as optimally low when ≤10%, good when 10%<to ≤20%, reasonable when 20%<to <50%, and inaccurate when 50%.The MAPE was used as a metric in this study because its complement (1 – MAPE)directly reflects the model’s accuracy.

To examine the practical application of the models as an early warning system, a comparative analysis of alarm and epidemic thresholds was conducted for the test period. The methodology used by the QCESU is based on the Philippine Department of Health’s Dengue Manual of Procedures and defines two key thresholds [60,61]. The alert threshold represents the average dengue caseload anticipated for the current epidemiological period and is established using a moving average of the current and preceding two years [60,61]. The epidemic threshold is then set at two standard deviations above this alert threshold [60,61]. Notably, the Philippine Department of Health’s manual does not define a model-based “alarm threshold.” For the comparative analysis, we replicated the epidemic threshold used by the QCESU. We compared it with the model-derived alarm and epidemic threshold. In this study, the alarm threshold was defined as the upper boundary of the 85% prediction interval corresponding to the point forecast +1-standard deviation of the forecast error for each week in the horizon, while the epidemic threshold was the upper boundary of the 95.5% prediction interval.

## Results

**Fig 2:**
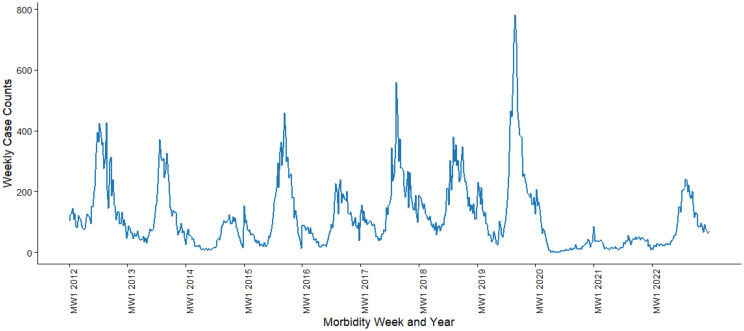
Weekly dengue case counts from 2012 to 2022 in Quezon City, Philippines. The X-axis represents the Morbidity Week; the Y-axis represents the weekly case count.

**Fig 3:**
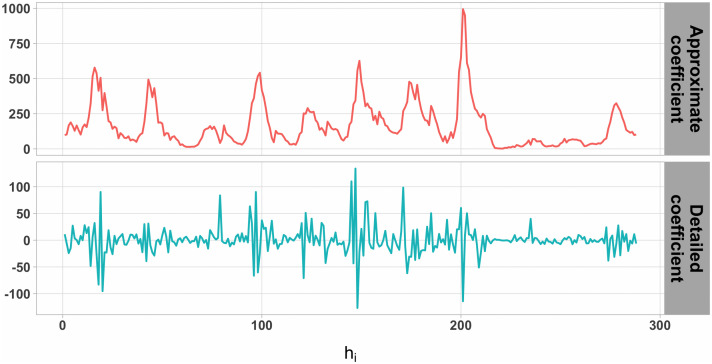
Approximate (cA, top) and detail (cD, bottom) coefficients after DWT. The decomposition at level 1 results in a time series for both cA and cD with approximately half the number of data points of the original series (*n = 269*) as the result of downsampling due to filtering; the X-axis represents to the horizon (data point) in the resulting time series of coefficients, *i,* Y-axis shows the coefficient.

The plotted weekly case counts from 2012 to 2022 are presented in Fig 2. The data shows clear seasonality, with cases typically increasing between MWs 25–40 every year. The highest number of cases occurred in MWs 34 and 36 of 2019, while the reported number of cases was markedly low during the Years 2020–2022. The DWT segregated the time series of weekly case counts into its cA and cD components, shown in Fig 3. The plot of the cA likewise shows the seasonality of dengue.

**Fig 4:**
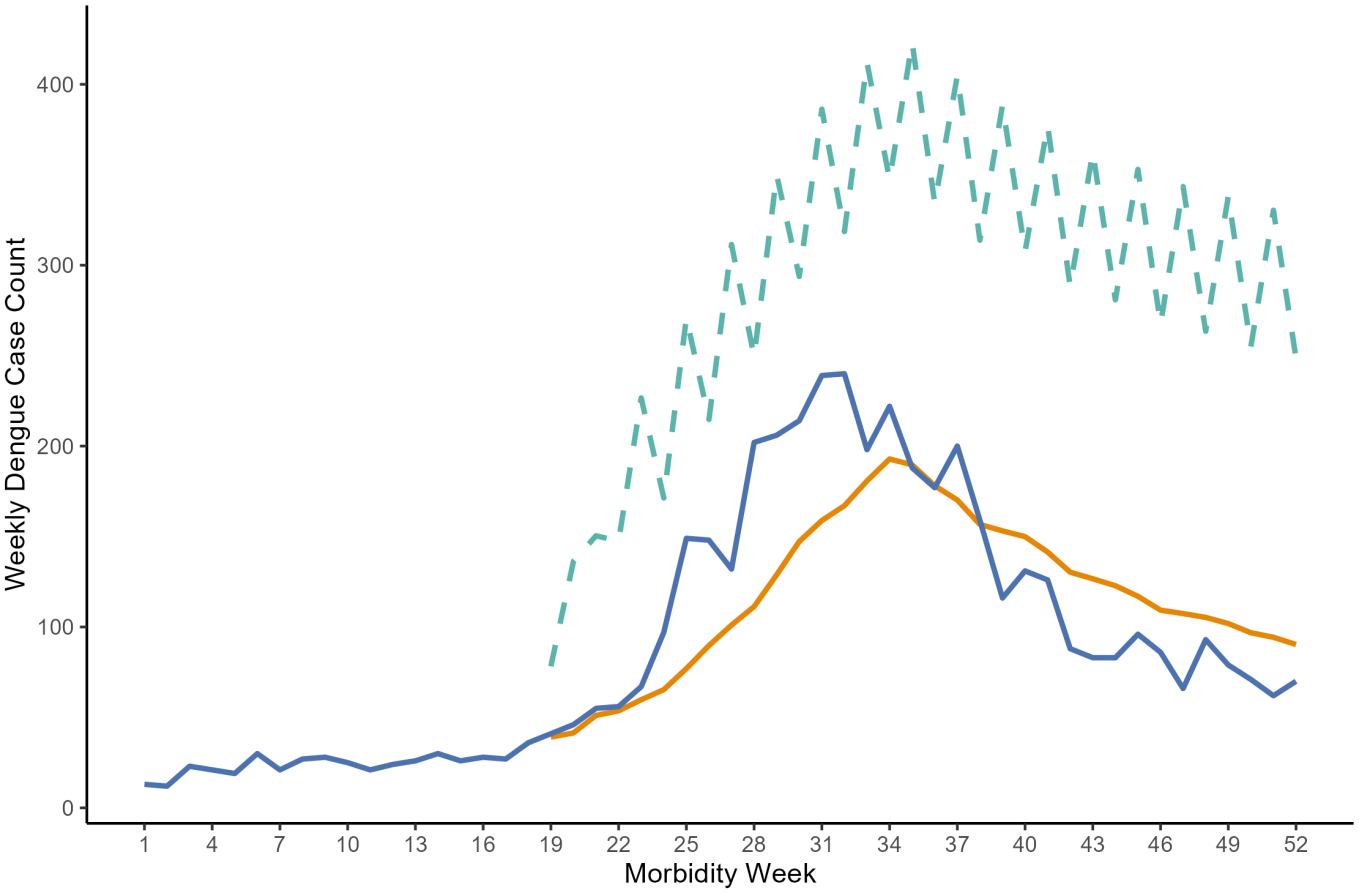
Out-of-sample with its 95.5% interval limit from the DWT-SARMA model and reported weekly case counts, 2022. The blue line represents reported weekly case counts from MW 1–52 of 2022; the orange line presents the point estimates of the forecasts from the DWT-SARMA model; the green line shows the 95.5% prediction interval (PI) estimate of the forecasts.

**Fig 5:**
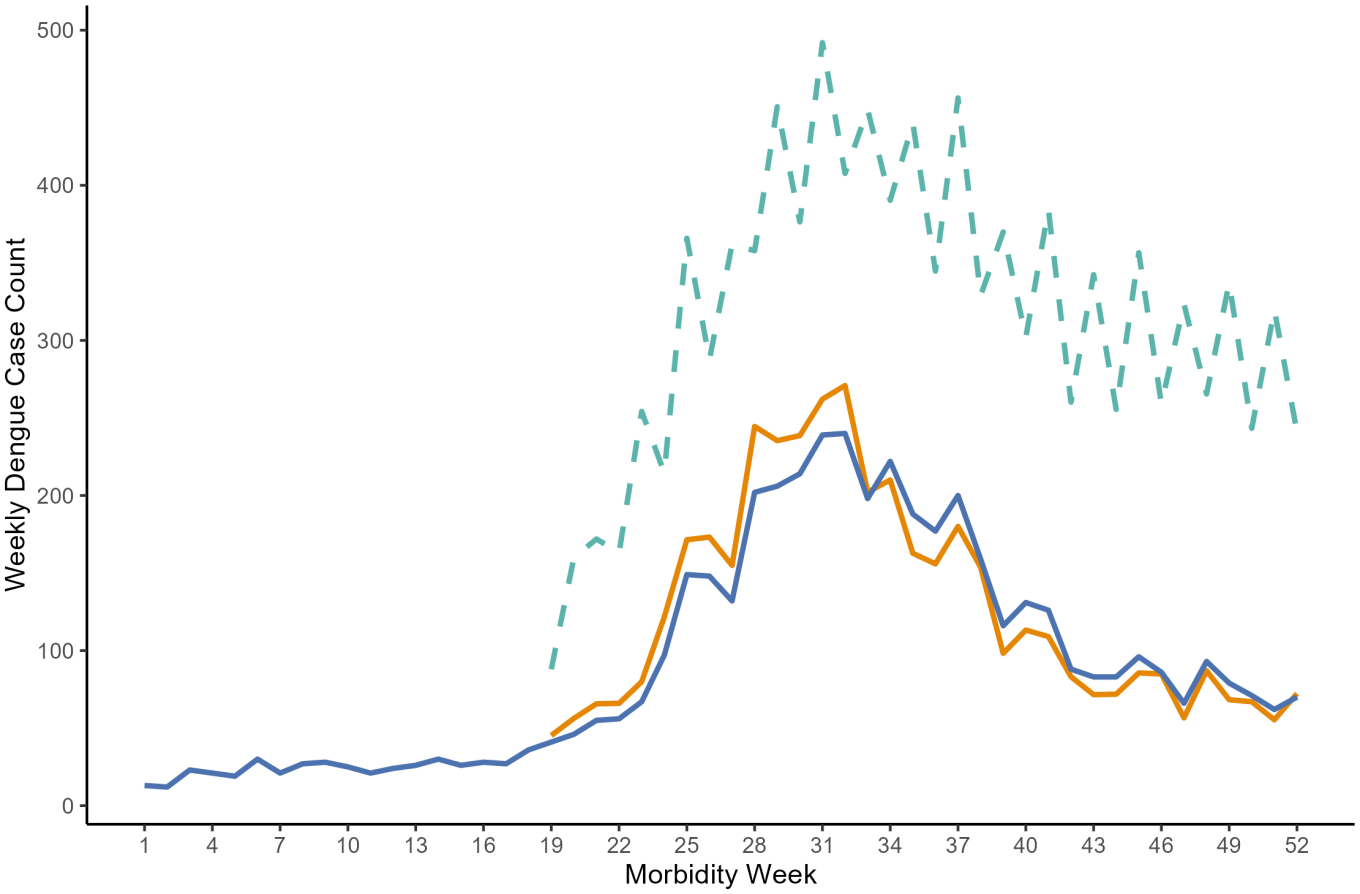
Out-of-sample with its 95.5% interval limit from the DWT-SARMA-LSTM model and reported weekly case counts, 2022. The blue line shows reported weekly case counts from MW 1–52 of 2022; the orange line presents the point estimates of the forecasts of the DWT-SARMA-LSTM model; the green line shows the 95.5% prediction interval (PI) estimate of the forecasts.

The best-fitting models were an ARMA (0,1) for the cD component, and a SARMA (2,2) x (1,1)_26_ for the cA component. The plots of the forecasted number of cases from the DWT-SARMA and DWT-SARMA-LSTM models during MWs 19–52 of 2022 are shown in Figs 4 and 5, respectively. A visual comparison of the out-of-sample forecasts reveals a clear difference in performance between the two hybrid models. The DWT-SARMA model failed to capture the peaks and troughs of the weekly case counts despite a reasonable MAPE of 26%. On the other hand, out-of-sample forecasts from the DWT-SARMA-LSTM model closely resemble the trend and spikes of the original data. In addition, the MAPE of the DWT-SARMA-LSTM model was 12.4%, indicating that the forecast accuracy level is 87.6%. Within an early warning system, the forecasted weekly case counts represent the expected number of cases, or the ‘endemic channel,’ while the upper limit of the 95.5% prediction interval acts as the ‘alarm threshold.’

Inspection of the MAPE for every 4-week increase in forecast horizon (Table 1) showed that the DWT-SARMA-LSTM model yielded more consistent forecast accuracy than the DWT-SARMA model, with forecast errors ranging from 12.4% to 18.3%.

**Table 1:** Mean Absolute Percentage Error (MAPE) of the DWT-SARMA and DWT-SARMA-LSTM for every 4-week increase in forecast horizon.

Number of forecast horizons	MAPE	
	DWT-SARMA	DWT-SARMA-LSTM
**4 weeks**	6.45%	17.48%
**8 weeks**	19.61%	18.33%
**12 weeks**	24.49%	17.56%
**16 weeks**	23.73%	15.05%
**20 weeks**	19.87%	13.97%
**24 weeks**	21.01%	13.64%
**28 weeks**	23.35%	13.10%
**32 weeks**	24.85%	12.71%
**34 weeks**	25.77%	12.37%

**Fig 6:**
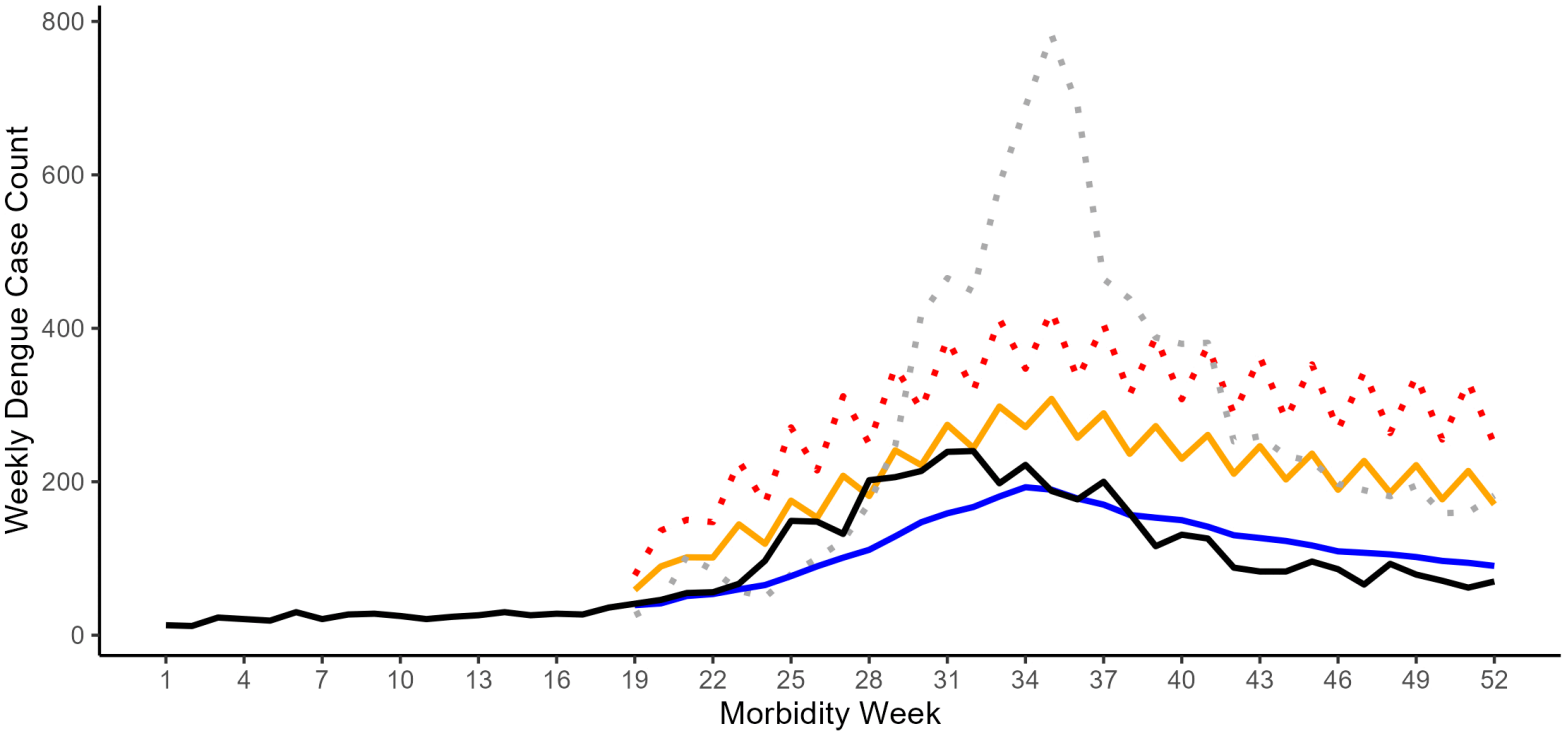
Comparison of alarm and epidemic thresholds for 2022 (DWT-SARMA model). The figure shows the reported weekly dengue cases (solid black line), DWT-SARMA forecast (solid blue line), model-derived alarm threshold (solid orange line), model-derived epidemic threshold (dotted red line), and traditional alert threshold calculated from the 3-year historical moving average (dotted grey line).

**Fig 7:**
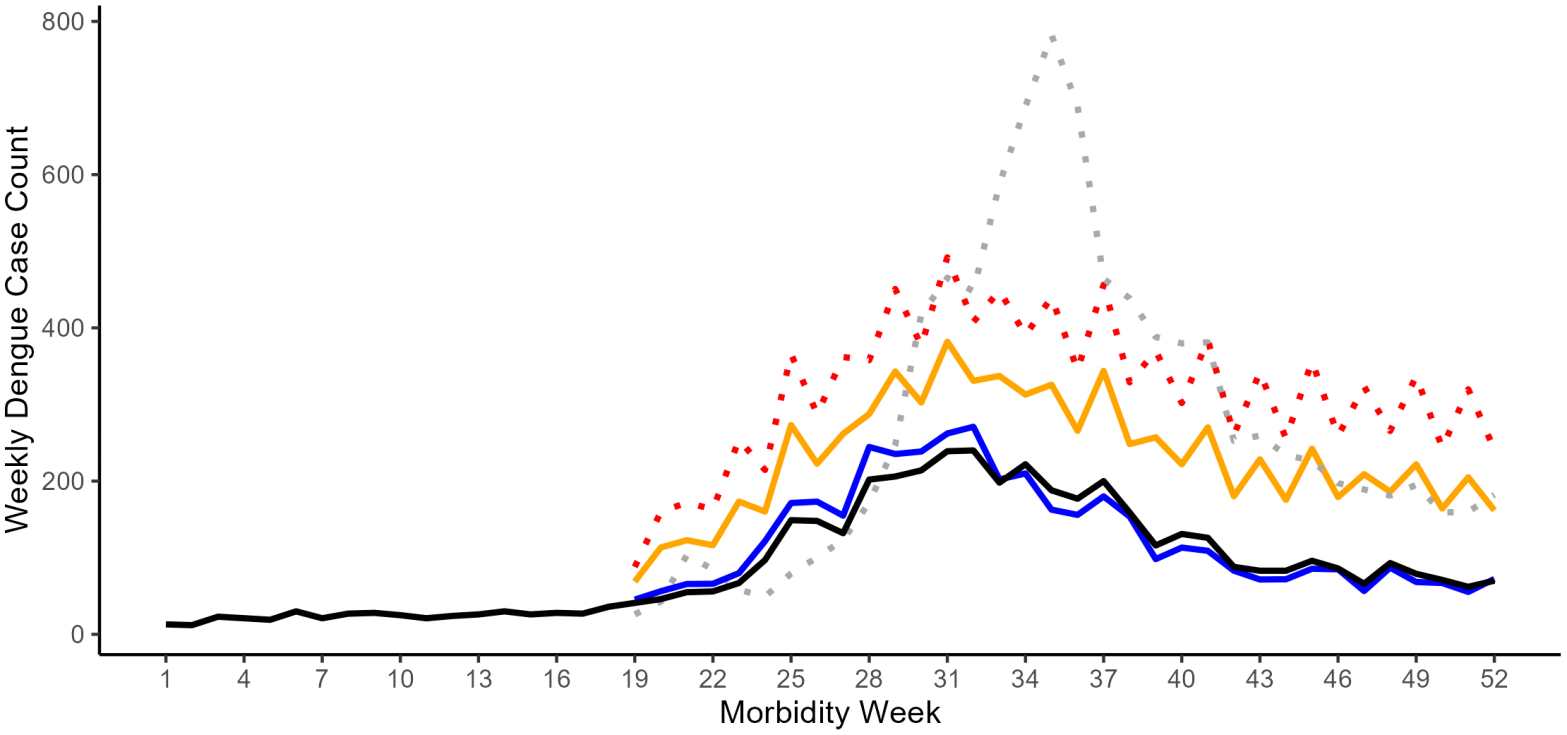
Comparison of alarm and epidemic thresholds (DWT-SARMA-LSTM model). The figure shows the reported weekly dengue cases (solid black line), DWT-SARMA-LSTM forecast (solid blue line), model-derived alarm threshold (solid orange line), model-derived epidemic threshold (dotted red line), and traditional alert threshold calculated from the 3-year historical moving average (dotted grey line).

The comparative analysis of threshold behavior shown in Figs 6 and 7 revealed distinct temporal patterns between model-derived thresholds and those computed from historical moving averages. During epidemiological MW 19–25 and MW 42–52, both the DWT-SARMA and DWT-SARMA-LSTM models produced alarm and epidemic thresholds that exceeded the epidemic threshold derived from the moving average method. In contrast, during the mid-season period (MW 26–41), the model-derived thresholds were markedly lower than the historical benchmark. These threshold shifts were consistent across both modeling approaches and suggested a divergence in how static and dynamic methods respond to recent transmission patterns. The elevated thresholds in early and late-season weeks coincided with periods of short-term volatility in weekly dengue case counts, while the lower thresholds in mid-season weeks aligned with a period of suppressed transmission. Additionally, the model-derived thresholds exhibited jagged contours across the forecast horizon, reflecting week-to-week variability in prediction uncertainty. This irregularity was most pronounced during volatile transmission periods, consistent with the influence of high-frequency components and residual error dynamics.

## Discussion

We forecasted weekly dengue case counts using the DWT-SARMA and DWT-SARMA-LSTM models during MWs 19–52 of 2022 (*h* = 34 weeks). While the DWT-SARMA model failed to capture the peaks and troughs of weekly case counts, the DWT-SARMA-LSTM model more closely matched the actual observation in the test data, achieving a lower MAPE of 12.4%, corresponding to an accuracy of 87.6%. The DWT-SARMA-LSTM model remained consistent over 4-MW forecast intervals, with MAPEs within the range for good forecasts. The alignment between the forecasted and observed fluctuations in weekly case counts highlights the model’s potential as an effective early warning system. This weekly predictive granularity is critical for timely detection of transmission shifts and for guiding public health actions that follow standard surveillance cycles.

The superior performance of DWT-SARMA-LSTM likely reflects LSTM’s ability to capture non-linear patterns and adapt to noise and shifting trends. However, LSTM models are computationally intensive due to recurrent matrix operations and careful hyperparameter tuning. To improve feasibility, we fixed look-back points and batch size based on SARMA residuals and implemented early stopping, reducing training time and making the model more practical for resource-constrained settings.

In the Philippines, outbreak detection uses epidemiologic criteria, in which a 3-year monthly moving average functions as both endemic channel and alert threshold. A 2-SD rise or a 50–60% increase from the baseline signals excess cases in current national practice [60,61]. Although computationally simple, this method assumes independence of observations and is sensitive to extreme values. Brady et al. (2015) emphasized the importance of critically evaluating which years are included in baseline calculations, because inclusion of periods with unusually high or low case counts may distort threshold sensitivity and delay detection [9]. Our models included visually identified years with elevated case counts. Despite this, our models produced forecasts with good to reasonable accuracy—even when tested during low-incidence periods during the COVID-19 pandemic—demonstrating robustness to baseline inflation and the ability to detect subtle transmission shifts.

A key distinction between our model and traditional approaches lies in the computation of thresholds. Effective dengue forecasting and outbreak detection requires accounting for long-term trends and seasonality, as well as irregular fluctuations, which may signal changes in transmission patterns. Approaches using averages generate smooth curves that approximate trend and seasonality, but they often overlook short-term volatility and emerging anomalies. In contrast, our model generates thresholds incorporating weekly forecast errors, resulting in jagged shapes. This jaggedness represents uncertainty and persists after peak weeks due to short-term fluctuations. By utilizing the cD component of the wavelet decomposition, the model captured these irregular patterns. Although the jagged appearance is visually less stable, the model-derived thresholds offer a more responsive representation of uncertainty, allowing the model to adjust to transient shifts in dengue transmission patterns that static methods may miss.

The difference between the model-derived thresholds and the historical moving average has implications for surveillance and response. During MWs 19–25 and 42–45, the DWT-SARMA-LSTM thresholds were higher than the moving average threshold, reducing false positives by filtering noise but potentially delaying outbreak detection during early transmission periods. In contrast, during MW 26–41, thresholds were markedly lower than the moving average threshold, increasing sensitivity to deviations when incidence was suppressed. This resulted from including 2019 in the moving average calculation, a year with relatively high dengue activity before the COVID-19 pandemic. The pandemic suppressed dengue transmission and disrupted healthcare capacity, altered treatment-seeking behavior, and reporting, resulting in low incidence during 2020–2022. These changes challenge the assumptions of classic time-series models such as SARIMA and ARIMA that rely on stationarity and consistent temporal relationships. In this study, we retained all available training data, including pandemic-period lows, and the DWT-SARMA-LSTM model still reflected fluctuations in observed case numbers. Its weekly updating mechanism and hybrid structure allow adaptation to evolving epidemiologic conditions and accurate forecasts. This adaptability is particularly valuable in post-pandemic contexts, where average-based thresholds may no longer reflect current transmission dynamics.

While this study validates the model using historical data, the DWT-SARMA-LSTM framework is designed for prospective forecasting and can be applied to future outbreaks. The model only requires weekly case counts, which are routinely collected in the surveillance system, as input and generates multi-step forecasts by learning temporal patterns. When future observations are not yet available, the model uses its own predictions recursively to simulate forward trajectories over longer horizons. This autoregressive capability, combined with responsiveness to short-term fluctuations, supports real-time surveillance and early warning applications. Forecast accuracy depends on input quality and reporting stability, yet the model enables weekly updates that incorporate new information and adapt to evolving transmission patterns.

This study advances existing literature by forecasting dengue at weekly intervals, an operationally important but underexamined timescale in the Philippines. Prior studies relied on monthly surveillance data, which obscures rapid changes in transmission. Weekly data, although more volatile and sparser, offers earlier signals for outbreak detection and supports timely resource allocation. Our study establishes a forecasting framework that addresses the pronounced short-term variability in weekly series, ensuring more reliable model performance when faced with anomalies [62]. By addressing these challenges through a hybrid model, we demonstrate that dependable weekly forecasting is achievable and operationally relevant, reaching 88% accuracy.

Our approach differs from previous studies in three major respects. First, unlike Necesito et al. (2021) and Ligue & Ligue (2021), who applied monthly case numbers, we use weekly data to enhance temporal sensitivity and facilitate earlier intervention [15,43]. Weekly monitoring enables earlier detection of transmission spikes and more timely public health responses, whereas monthly aggregation may obscure short-term anomalies and delay critical interventions. Second, we preserve both low-frequency (cA) and high-frequency (cD) wavelet components, whereas Necesito et al. (2021) excluded cD, removing informative short-term fluctuations for outbreak detection [15]. Third, the hybrid DWT-SARMA-LSTM architecture unifies SARMA for seasonality with LSTM for nonlinear residual learning. This structure strengthens adaptability to abrupt changes and improves accuracy, while parameter simplification supports implementation in resource-limited settings.

To our knowledge, this is the first work in the Philippines to forecast weekly dengue incidence without climate variables. Although climate affects transmission [63–65] and is frequently used in high-performing models [18,21,23,42,46], our method provides a practical alternative where climate data availability is limited. It aligns with national initiatives promoting machine learning and AI integration in public health [66].

The DWT-SARMA-LSTM framework is extendable to other infectious diseases. DWT separates trend and irregularity, SARIMA captures lagged linear relationships, and LSTM retains long-term dependencies and data-driven features. This integration improves accuracy and robustness for diseases with complex dynamics, including influenza, tuberculosis, malaria, and leptospirosis, particularly in areas with established surveillance but limited access to climate data.

This study has several limitations. First, the inclusion of periods with unusually high or low weekly case counts affected the DWT-SARMA accuracy, although the hybrid DWT-SARMA-LSTM model performed consistently. Future studies should test sensitivity to potential data singularities. Second, testing occurred in a period without a formally declared outbreak, limiting assessment of sensitivity, specificity, and timeliness for outbreak detection. Third, our focus was on epidemiologic thresholds, whereas public health emergencies depend on system capacity. Fourth, underdiagnosis and underreporting may have influenced model training quality. Finally, evaluation under outbreak conditions and the inclusion of external variables such as climate, vegetation indices, population density and mobility, and mosquito population and breeding site density may enhance performance. Future research may also assess advanced machine learning strategies, including attention mechanisms, to improve long-term dependency modeling and interpretability.

In conclusion, the DWT-SARMA-LSTM model is a robust and adaptable framework for forecasting weekly dengue cases and establishing dynamic alert thresholds using univariate surveillance data in Quezon City, Philippines. By combining wavelet decomposition, seasonal autoregressive modeling, and deep learning residual correction, the approach captures both linear and nonlinear transmission dynamics, addressing weaknesses of traditional models and adjusting to post-pandemic trends. Its reliance on routinely collected surveillance data and operational practicality underscores its potential for real-time early warning in resource-constrained environments. Continued validation in diverse geographic settings and disease contexts is warranted.

## Supporting information

S1 Text Supplementary material. The file contains additional information about the methodology. Further explanations on the following steps are listed in the file: Data Source and Preprocessing, Discrete Wavelet Transform, SARMA Modeling, LSTM Modeling of SARMA Residuals, Forecast Reconstruction, Accuracy Evaluation.
(DOCX)
