# Supplementary material for: Advancing outbreak detection: Hybridizing machine learning with wavelets for weekly dengue case forecasting
Source: PLoS Negl Trop Dis. 2026 Jul 9;20(7):e0014444. doi: 10.1371/journal.pntd.0014444 (PMC13349185; doi:10.1371/journal.pntd.0014444)
Supplement: S1 Text — The file contains additional information about the methodology. Further explanations on the following steps are listed in the file: Data Source and Preprocessing, Discrete Wavelet Transform, SARMA Modeling, LSTM Modeling of SARMA Residuals, Forecast Reconstruction, Accuracy Evaluation. (DOCX) [file pntd.0014444.s001.docx]

**Supplementary Material**

A. Data Source and Preprocessing

- **Data origin**: Weekly dengue case data (January 2012–December 2022) were obtained from the Quezon City Epidemiology and Surveillance Unit (QCESU), Philippines. These proprietary data are not publicly available.
- **Temporal aggregation**: Daily case records were aggregated into weekly counts based on the Philippine Integrated Disease Surveillance and Response (PIDSR) “Morbidity Week” definition.
- **Completeness check**: No missing weekly records were identified; therefore, no imputation was performed.
- **Variables used**: Only weekly case count (n) was used for modeling. No external covariates (e.g., climate, vector indices) were incorporated.

B. Discrete Wavelet Transform (DWT)

- **Purpose**: DWT was used to decompose the weekly dengue time series into low-frequency (approximate, cA) and high-frequency (detail, cD) components. This decomposition enables the separation of long-term trends from short-term fluctuations and noise, improving interpretability and forecast performance of time series models.
- **Wavelet filters and families:** In wavelet analysis, a *filter* refers to a mathematical function used to extract specific frequency components from a signal. Filters are applied in pairs—low-pass filters capture smooth, large-scale patterns (approximation), while high-pass filters isolate rapid, localized variations (details). A *filter family* is a set of wavelets with shared structural properties such as symmetry, compact support, and vanishing moments, which influence how effectively features in the data are represented.

We selected the Daubechies wavelet family because it is effective for analyzing non-stationary time series with sharp transitions and seasonal patterns, which are typical features of infectious disease data. Daubechies wavelets are orthogonal and compactly supported, allowing accurate representation of both smooth seasonal trends and abrupt changes without artificial oscillations. These characteristics make them well suited for epidemiological signals that display seasonal cycles and outbreak spikes.

The term *db3* refers to a third-order Daubechies wavelet with three vanishing moments, enabling exact representation of polynomials up to degree two and supporting modeling of quadratic trends in the data. db3 provides an effective balance between time and frequency localization, making it suitable for decomposing weekly dengue case counts that exhibit gradual seasonal trends alongside short-term anomalies. Among the tested filter-level configurations, db3 at level 1 achieved the lowest Root Mean Square Error (RMSE) during reconstruction of the original time series and was therefore selected for model development.

- **Decomposition level**: Level 1 was selected because it minimized RMSE between the reconstructed and original series. This level retained adequate temporal resolution while allowing effective separation of dominant signal components.
- **Downsampling**: Level 1 DWT reduced the number of data points by half (from 538 to 269 in the training set).

C. SARMA Modeling

- **Model selection**: The auto.arima() function from the statsmodels Python package independently identified optimal SARMA models for cA and cD.
- **Criteria**: Models with the lowest Akaike Information Criterion (AIC) were selected.
- **Best-fitting models**:
  - cA: SARMA(2,2) × (1,1)[26]
  - cD: ARMA(0,1)
- **Forecast horizon**: 17 weeks (matching the downsampled test set size).

D. LSTM Modeling of SARMA Residuals

- **Purpose**: To capture non-linear patterns not modeled by SARMA.
- **Input preparation**:
  - Residuals were normalized to [0,1] using min-max scaling.
  - Look-back window determined via autocorrelation and partial autocorrelation analysis.
- **Architecture**:
  - Two-layer LSTM: one sequential layer and one dense output layer.
  - Optimizer: Adam (learning rate = 0.001)
  - Loss function: Mean Squared Error (MSE)
  - Batch size: 4
- **Hyperparameter tuning**:
- Epochs: Tested from 10 to 300 in increments of 10; 90 epochs selected based on lowest mean error and narrowest error range.
- Neurons: Tested from 1 to 20; 3 neurons for cA residuals and 1 neuron for cD residuals selected.
- Early stopping: Patience = 2

E. Forecast Reconstruction

- **Final forecast**: Forecasted residuals from LSTM were added to SARMA forecasts for cA and cD.
- **Inverse DWT**: Combined forecasts were reconstructed into weekly case counts using inverse DWT.
- **Prediction intervals**:
- Alarm threshold: Point forecast + 1 SD (85% interval)
- Epidemic threshold: Point forecast + 2 SD (95.5% interval)

F. Accuracy Evaluation

- **Metric**: Mean Absolute Percentage Error (MAPE)
- **Interpretation**:
  - <10%: Optimally low
  - 11–20%: Good
  - 21–50%: Reasonable
  - 50%: Inaccurate

**Forecast horizons**: MAPE was computed for every 4-week increment up to 34 weeks.
