# Supplementary material for: Advancing outbreak detection: Hybridizing machine learning with wavelets for weekly dengue case forecasting
Source: PLoS Negl Trop Dis. 2026 Jul 9;20(7):e0014444. doi: 10.1371/journal.pntd.0014444 (PMC13349185; doi:10.1371/journal.pntd.0014444)
Supplement: Attachment — Submitted filename: Review_PLOSNEG.pdf [file pntd.0014444.s002.pdf]

Review Manuscript Number: PNTD-D-25-00682 PLOS NTD

Advancing Outbreak Detection: A Hybrid Model for Weekly Dengue Case Forecasting

The present study aims to present an alternative method for forecasting the number of dengue cases, using as a case study a 10 year dataset of weekly dengue case counts in Quezon City, Philippines. The study also compares the proposed method with a previously developed hybrid model DWT-LSTM applied to monthly data.

The main objectives of the work are to:

1. Demonstrate that the proposed hybrid model outperforms previous approaches.

2. Define an early warning system for dengue outbreaks.

While the study explores an interesting idea, it is unclear whether it falls within the scope of PLOS Neglected Tropical Diseases, for the following reasons:

1. Case studies are only considered when they present novel insights.

2. It is not clearly established that the application of the DTW-LSTM model to weekly dengue data constitutes a novel contribution or the novelty of DWT-SARMA-LSTM.

The manuscript requires substantial revision in terms of English grammar and scientific writing. Although a few issues are noted here, many others will be not highlight here. Moreover, the content would benefit from significant rephrasing to improve readability. Several statements throughout the manuscript are also unclear or insufficiently supported (see Issue 1, for example).

The overall idea is promising, and the study might yield more meaningful insights if the authors extended their discussion and addressed several methodological and conceptual issues.

A major concern lies in the clarity of the methodology. Given the broad scope of the journal and its diverse readership, the methods section should be described more clearly and systematically. For instance, Figure 1 and the accompanying text do not adequately explain the hybrid model construction or which variables were used as inputs, for instance. Furthermore, the integration and interaction between the components of the hybrid model are not clearly articulated.

Therefore, I do not recommend this manuscript **as it is** for publication in **PLOS Neg**. More detailed comments supporting this decision are provided below.

Major Issues:

Issue 1.

The authors state: "The proposed method effectively tackled the complexities of short-term fluctuations and sparseness of data."

However, it is unclear how this problem was addressed in the manuscript. The use of weekly data instead of monthly data appears to be due to the temporal scale available in the dataset, rather than as a deliberate methodological choice to address data sparsity or fluctuations. The authors are encouraged to explain in greater detail how their proposed method specifically deals with short-term variability and sparse data, and provide supporting evidence or analysis.

Issue 2.

Although the manuscript applies the proposed method to the Quezon City dataset, this is not mentioned in the title, abstract, or introduction, which significantly limits the contextual framing of the study.

Furthermore, in the only section where the study area and data source are briefly mentioned, the country is omitted. The authors should explicitly state that Quezon City is located in the Philippines. This is a basic but important detail for the reader to understand the geographical and epidemiological context of the study.

Issue 3.

Regarding the dataset, the authors do not clearly specify the source of the data, whether it is publicly available (open-source) or proprietary. Additionally, there is no description of the type of data used, nor of the preprocessing steps undertaken prior to modeling.

It is essential that the manuscript documents:

1. How the data was collected and prepared for analysis.

2. Whether any missing data handling, normalization, transformation was performed.

3. What software or packages were used for the analysis (though some are mentioned, the methodological flow remains difficult to follow).

The manuscript should also clarify how the hybrid modeling approach was implemented, especially how the different components (e.g., SARMA and LSTM) were linked. Without these methodological details, the reproducibility and transparency of the study are compromised.

Please include proper references for the techniques employed, to ensure clarity and reproducibility for readers who may wish to replicate or build upon this work.

Issue 4.

The authors state: "The unreliability and heterogeneity of performance of commonly used methods for determining an outbreak can be attributed to the complexity of the disease transmission dynamics, as four distinct dengue

serotypes have extensive genetic diversity within each serotype and are driven by diverse environmental factors."

This statement is somewhat unclear and could be misinterpreted. As written, it suggests that the geographic distribution of the four dengue serotypes is directly driven by environmental or climatic factors, which may not be entirely accurate or universally supported. If this is the intended meaning, the authors should provide appropriate references to substantiate this claim.

Alternatively, the sentence should be rewritten for clarity, clearly distinguishing between the genetic diversity of serotypes, the complexity of transmission dynamics, and the role of environmental factors in influencing outbreak patterns. Ambiguity in this type of statement can lead to confusion, especially for interdisciplinary readers.

Issue 5.

The authors state as their first objective: "We aim to assess the performance of DWT-SARMA and DWT-SARMA-LSTM models in forecasting the weekly number of dengue cases."

While it is clear that the manuscript attempts to address this goal, the novelty of the hybrid model remains unclear due to the lack of methodological detail. Specifically, the manuscript should explain in what ways the proposed hybrid approach differs from prior work, such as that of Necesito and Kim (Vol. 52, No. 4, 2021), who reportedly used a similar methodology on monthly data.

In my view the current manuscript should clarify whether the novelty lies in the enhanced model integration (and how this was coupled) rather than purely the use of weekly data.

Moreover, they should focus and clearly describe how the SARMA component was incorporated. Highlight what distinguishes this study from this previous work, particularly with regard to how the hybrid model is constructed and applied.

By emphasizing these aspects, the authors will better justify their methodological contribution.

Issue 6.

The authors state: "As a secondary objective, we will be exploring the potential of the univariate models in defining the level of the alarm thresholds for dengue using the discrete wavelet transform (DWT) method."

It was not clear to me how this second objective was addressed. The manuscript would benefit greatly from a more in-depth discussion of how the findings relate to practical public health applications, particularly regarding early warning systems for dengue outbreaks in Quezon City.

Given that the testing period includes data from 2022, the authors should take this opportunity to explore how their results could have contributed to real-time outbreak alerts or control measures. Additionally, the use of figures to illustrate the step-by-step of how this was addresses would strengthen the paper.

To improve transparency and reproducibility, I suggest including a more detailed description of the methods (e.g., DWT implementation, threshold setting, filter meaning/ description, / variable type and scale, and model training/testing procedure) in the supplementary material, so that readers do not need to rely on external sources to understand the methodology.

Issue 7.

The authors state: "DWT's ability to segregate trends, seasonality, and noise from the data enhances out-of-sample forecasting accuracy compared to traditional methods like autoregressive integrated moving average (ARIMA) and allows the reconstruction of the original data from these coefficients."

This claim requires proper literature support. For example, the following study may be relevant: Chen, X., Moraga, P. (2025). Assessing dengue forecasting methods: a comparative study of statistical models and machine learning techniques in Rio de Janeiro, Brazil. Tropical Medicine and Health, 53(1), 52. https://doi.org/10.1186/s41182-025-00723-7

Issue 8.

Figure caption, interpretation and legends need improvement. Based on the expected number of data points, the DWT coefficients figure should display 532 points, yet it appears to show only around 300. This discrepancy needs clarification.

Additionally, figure legends and captions, particularly for Figure 2, should be more informative and self-contained. Captions should include, what the figure shows (e.g., original time series, decomposed components) and time frame or units if applicable.

Also Figure 1 is not self explicative and does not help in understanding the methodological approach described in text. Improved visual communication is essential for both expert and non-expert readers to follow the analysis.

Issue 9.

A broader conceptual question arises: is the study truly about forecasting, or is it more about retrospective model validation using past data?

The manuscript would benefit from a discussion on whether this approach could be realistically used to forecast future outbreaks (e.g., for 2025 or 2026). Specifically:

- What inputs would be required to produce such forecasts?
- How would this method handle the absence of future data for model input?
- Could an early warning system realistically be given using this method?

Clarifying the applicability and limitations of the proposed models in forecasting (beyond retrospective testing) would enhance the relevance and practical value of the study.

Issue 10.

The authors state: "We built two hybrid models—a DWT-SARMA model and a DWT-SARMA-LSTM model."

Given that a similar approach (DWT-SARMA) was used by Necesito and Kim (2021) with monthly incidence data, the manuscript would benefit from a detailed comparison between the previous and current work.

Specifically, the authors should highlight:

- What has changed in the model structure or implementation.
- Why the transition from monthly to weekly data improves the model?
- How the SARMA was coupled to the existing method.

Clearly articulating these points would help justify the novelty and contribution of the current study.

Issue 11.

A critical discussion is missing regarding the statement: "The forecasted mean number of cases was used in defining the alarm and outbreak thresholds, respectively."

This process is essential to the utility of the model for public health decision-making, yet it is not thoroughly explained or justified in the manuscript. The following question should be addressed:

- How were the alarm and outbreak thresholds defined? Can be defined for a real forecast in 2025?

Expanding this discussion will strengthen the paper's claims regarding its potential use in early warning systems and make the findings more actionable for public health authorities.

Minor Issues:

1. Study Area Description

The study area section does not clearly state that the analysis is a case study conducted in the Philippines.

2. Figure Caption

In the caption of Figure 2, the term "Morbidity Week" is used. However, in the Materials and Methods section, the data is described as weekly dengue case counts. The terminology should be consistent, and the manuscript should clearly define:

- The source of the data and variables.
- The temporal resolution (e.g., "morbidity week" vs. "calendar week").
- The variables included.
- Any preprocessing, transformation applied.

3. Line 113

There is an excess space that should be removed for consistency in formatting.

4. Line 140

Another spacing issue is present. Additionally, check the reference citation in the text. It appears in many occasions immediately after a final period. Be-

sides, Necesito was cited as Necessito once.

5. Abbreviation Usage

The abbreviations “cA” and “cD” introduced around line 150 should continue to be used consistently throughout the manuscript after they are first defined. Avoid reintroducing or switching back to the full term unnecessarily.

6. Abstract

To be revised:

The authors affirm first: ”We determined the forecast accuracy of the **two models** using the mean absolute percentage error.” That is, they assume the readers knowledge of know two models before presenting them. Although later they do that.

7. Data clarification

Data in abstract is said to be from 2011 to 2019, while in data section 2012 to 2022.
