# Supplementary material for: Advancing outbreak detection: Hybridizing machine learning with wavelets for weekly dengue case forecasting
Source: PLoS Negl Trop Dis. 2026 Jul 9;20(7):e0014444. doi: 10.1371/journal.pntd.0014444 (PMC13349185; doi:10.1371/journal.pntd.0014444)
Supplement: Attachment — Submitted filename: Response letter_1107FINAL.docx [file pntd.0014444.s004.docx]

Response to Reviewers

Manuscript Title: *Advancing Outbreak Detection: A Hybrid Model for Weekly Dengue Case Forecasting*
Manuscript Number: PNTD-D-25-00682
Journal: PLOS Neglected Tropical Diseases

Dear Editors and Reviewers,

We sincerely thank you for your thoughtful and constructive feedback on our manuscript. We appreciate the opportunity to revise our work and have carefully addressed each comment raised. Below, we provide a point-by-point response, indicating how we have revised the manuscript accordingly.

Comments from Reviewer 1

Major Issues

1. Clarify how the model addresses short-term variability and sparse data

Response:
*We appreciate this observation. We have revised the manuscript to clarify that the use of weekly data was both a methodological choice at lines 200–203. We also clarified that weekly data introduces short-term fluctuations and sparsity, which our hybrid model is designed to handle at lines 406–414. DWT isolates high-frequency components (cD) (lines 220–221), while the LSTM captures nonlinear residual patterns (lines 241–242). Coupling these methods enabled the model to overcome the challenges in weekly data. Further explanation is now included in the Discussion at lines 440–441, 449–451, and 472–474. Supporting evidence is provided through the model’s performance across forecast horizons shown in Table 1 and Figure 5.*

2. Explicitly state the study location and country
Response: *We revised the Abstract, Introduction, Results and Discussion to explicitly state that the study was conducted in Quezon City, Philippines. These updates appear at lines 38, 58, 174, 179, 304 and 493.*

3. Clarify data source, preprocessing, and software used
Reviewer comment: *The manuscript lacks detail on data handling and software.*

*Response: We have expanded the Data Acquisition and Preprocessing and Statistical Analysis sections to provide a more systematic description of the dataset, preprocessing steps (e.g., aggregation, normalization), and software packages used at lines 190–210. It was also explicitly stated that the data is proprietary and not publicly available at line 198. We now describe in detail how the SARMA and LSTM components were integrated, including the flow of residuals and the reconstruction process. References for all libraries and techniques have been added to ensure reproducibility at lines 210, 218, 232, 247, and 258.*

4. Clarify statement on dengue serotypes and environmental factors
Reviewer comment: The statement may imply that serotype distribution is driven by environmental factors.

Response: W*e revised the sentence to distinguish between genetic diversity and environmental modulation:*

*“These dynamics are inherently complicated by the extensive genetic diversity across four distinct dengue serotypes and are additionally modulated by diverse environmental factors that affect transmission patterns .” (lines 112–114).*

5. Clarify novelty of the hybrid model
Reviewer comment: The manuscript should explain how the model differs from previous work.

Response: *We added a paragraph comparing our weekly DWT-SARMA-LSTM model to Necesito et al.’s monthly DWT-LSTM model at lines 454–464. We emphasized our integration of SARMA for linear structure and LSTM for residual refinement (lines 220–221, 440–441 and 472–474), as well as the operational relevance of weekly forecasts (lines 445 to 452).*

6. Expand discussion on alarm thresholds and public health application
Reviewer comment: The second objective is not clearly addressed.

Response: *We added the methods related to the second objective at lines 281 to 293. We added Figures 6 and 7 to illustrate how the models were used to define the alarm and epidemic thresholds, with the corresponding interpretation of results at lines 347–360. We also revised the Discussion to explain how model-derived thresholds were computed and compared to historical benchmarks (lines 406–433). A supplementary file explaining the steps, including DWT implementation, threshold setting, filter meaning/ description, / variable type and scale, and model training/testing procedure was also added.*

7. Support claim about DWT’s forecasting advantage
Reviewer comment: The claim requires literature support.

Response: *We cited Chen & Moraga (2025) and expanded the explanation of how DWT improves forecasting by separating trend and noise.*

8. Improve figure captions and clarify data point discrepancy
Reviewer comment: *Figure 3 appears to show fewer data points than expected.*

*Response: We clarified that level-1 DWT downsampling reduces the number of data points by half (lines 237–238 and 309–311). Figure 1 was also revised to improve the clarity of the method. Captions were revised to include time frame, units, and decomposition logic.*

9. Clarify whether the model is retrospective or prospective
Reviewer comment: The manuscript should discuss real-time applicability.

Response: *We added a paragraph in the Discussion explaining how the model can be used prospectively for future forecasts using current weekly data and autoregressive structure, as well as the limitations and recalibration needs at lines 435–443.*

10. Compare with Necesito et al. (2021) in more detail
Reviewer comment: The manuscript should highlight what has changed.

Response: *We expanded the comparison, emphasizing our use of SARMA for structured forecasting, LSTM for residual learning, and weekly data for finer temporal resolution at lines 454–464.*

11. Clarify how thresholds were defined and applied
Reviewer comment: The process is essential but underexplained.

Response: *We added formulas and definitions for alarm and epidemic thresholds based on prediction intervals at lines 281–293. We discussed how these thresholds could be used in real-time outbreak alerts at lines 435–443.*

Minor Issues

1. Study area description *Revised to state that Quezon City is in the Philippines clearly.*

2. Figure caption terminology
*“Morbidity Week” or “MW” is now consistently defined and used throughout.*

3–4. Formatting and citation placement
*Corrected spacing and citation formatting; “Necesito” spelling standardized.*

5. Abbreviation usage
*“cA” and “cD” used consistently after first definition.*

6. Abstract clarity
*Revised to introduce models before discussing results.*

7. Data range consistency
*Corrected to 2012–2022 across all sections.*

Comments from Reviewer 2:

1. Interpretation of Figures 4 and 5.

Reviewer: The key problem is the interpretation of figures 4 and 5.

Response: We corrected the labeling errors in Figures 4 and 5, thereby aligning the interpretation with the figures.

Other points:

1. The title seems to me not the best possible.
*Per the reviewer’s advice, we changed the title to
"Advancing Outback detection for Dengue: hybridizing machine learning with wavelets for forecasting".*

2. In the abstract, the accuracy of the classical approach must be represented as 74.2% and for the Hybrid model as 87.6%.

*We revised the abstract and used MAPE.*

Row 79: 595430
*We formatted the case counts.*
Row 148: cite the early papers on DWT, for example:

*We cited the work of Dorize et al.*
Row 161: Instead of "recently" it is better "in last 20 years".

*We replaced it with “in the last 20 years” (line 147).*
Row 114: there is an extra )
*We removed the extra parenthesis.*
Row 201: add that youbtefere to Philippines
*We mentioned the Philippines in the Introduction, Abstract, and Discussion.*
Figure 1: explain much better in the text, especially the rationale underlying the choice h=17
*We explained it at lines 236–239.*
Row 225: add a reference for the python module "pmdarima"
*We added the reference for pmdarima.*
Row 233: add a reference for Tensor flow
*We added the reference for it.*
Row 233: did you also use Keras ? If yes, add a reference to the book by Chollet
*We added the reference for Keras.*
Rows 277 to 279: use "Upper Panel:" and "Lower Panel"
*We used upper and lower panels in the figure captions as suggested.*
Row 241: add a reference for Adam
*We included the reference for Adam.*
Rows 244-245: the sentence " An experiment ..configuration" has no verb

*The methodology section was revised for improved clarity, which necessitated corresponding changes to the associated statement.*

We hope these revisions address the reviewers’ concerns and improve the clarity, rigor, and relevance of our manuscript. We are grateful for the opportunity to revise and resubmit, and we welcome any further feedback.
