# Supplementary material for: Advancing outbreak detection: Hybridizing machine learning with wavelets for weekly dengue case forecasting
Source: PLoS Negl Trop Dis. 2026 Jul 9;20(7):e0014444. doi: 10.1371/journal.pntd.0014444 (PMC13349185; doi:10.1371/journal.pntd.0014444)
Supplement: Peer review history

# Peer review: Decision Letter 0

**DOI:** 10.1371/journal.pntd.0014444.r001

**Authors:** Benn Sartorius, Maira Aguiar

11 Sep 2025

-->PNTD-D-25-00682

Advancing Outbreak Detection: A Hybrid Model for Weekly Dengue Case Forecasting

PLOS Neglected Tropical Diseases

Dear Dr. Nakamura,

Thank you for submitting your manuscript to PLOS Neglected Tropical Diseases. After careful consideration, we feel that it has merit but does not fully meet PLOS Neglected Tropical Diseases's publication criteria as it currently stands. Therefore, we invite you to submit a revised version of the manuscript that addresses the points raised during the review process.

Please submit your revised manuscript within 60 days Nov 10 2025 11:59PM. If you will need more time than this to complete your revisions, please reply to this message or contact the journal office at plosntds@plos.org. When you're ready to submit your revision, log on to https://www.editorialmanager.com/pntd/ and select the 'Submissions Needing Revision' folder to locate your manuscript file.

Please include the following items when submitting your revised manuscript:

* A rebuttal letter that responds to each point raised by the editor and reviewer(s). You should upload this letter as a separate file labeled 'Response to Reviewers'. This file does not need to include responses to any formatting updates and technical items listed in the 'Journal Requirements' section below.

* A marked-up copy of your manuscript that highlights changes made to the original version. You should upload this as a separate file labeled 'Revised Manuscript with Track Changes'.

* An unmarked version of your revised paper without tracked changes. You should upload this as a separate file labeled 'Manuscript'.

If you would like to make changes to your financial disclosure, competing interests statement, or data availability statement, please make these updates within the submission form at the time of resubmission. Guidelines for resubmitting your figure files are available below the reviewer comments at the end of this letter.

We look forward to receiving your revised manuscript.

Kind regards,

Maira Aguiar, PhD

Academic Editor

PLOS Neglected Tropical Diseases

Benn Sartorius

Section Editor

PLOS Neglected Tropical Diseases

Shaden Kamhawi

co-Editor-in-Chief

PLOS Neglected Tropical Diseases

orcid.org/0000-0003-4304-636XX

Paul Brindley

co-Editor-in-Chief

PLOS Neglected Tropical Diseases

orcid.org/0000-0003-1765-0002

**Additional Editor Comments:**

Please update the manuscript in accordance with the reviewers’ comments. Ensure that all revisions are clearly marked and that a detailed response to each reviewer’s point is provided.

**Journal Requirements:**

If the reviewer comments include a recommendation to cite specific previously published works, please review and evaluate these publications to determine whether they are relevant and should be cited. There is no requirement to cite these works unless the editor has indicated otherwise.

1) Please ensure that the CRediT author contributions listed for every co-author are completed accurately and in full.

At this stage, the following Authors/Authors require contributions: Yuta Hattori. Please ensure that the full contributions of each author are acknowledged in the "Add/Edit/Remove Authors" section of our submission form.

The list of CRediT author contributions may be found here: https://journals.plos.org/plosntds/s/authorship#loc-author-contributions

2) Please provide an Author Summary. This should appear in your manuscript between the Abstract (if applicable) and the Introduction, and should be 150-200 words long. The aim should be to make your findings accessible to a wide audience that includes both scientists and non-scientists. Sample summaries can be found on our website under Submission Guidelines:

https://journals.plos.org/plosntds/s/submission-guidelines#loc-parts-of-a-submission

**Reviewers' Comments:**

Reviewer's Responses to Questions

**Key Review Criteria Required for Acceptance?**

As you describe the new analyses required for acceptance, please consider the following:

**Methods**

-Are the objectives of the study clearly articulated with a clear testable hypothesis stated?

-Is the study design appropriate to address the stated objectives?

-Is the population clearly described and appropriate for the hypothesis being tested?

-Is the sample size sufficient to ensure adequate power to address the hypothesis being tested?

-Were correct statistical analysis used to support conclusions?

-Are there concerns about ethical or regulatory requirements being met?

Reviewer #1: The methodology is not clearly stated. See more in the attached document.

Reviewer #2: See my report

**********

**Results**

-Does the analysis presented match the analysis plan?

-Are the results clearly and completely presented?

-Are the figures (Tables, Images) of sufficient quality for clarity?

Reviewer #1: Figures are not self explained, missing legend and titles and axes names.

Data set is said to be 2011 to 2019 in abstract but in analysis 2012 to 2022.

Figure show 300 data points rather 532.

See more in attached document.

Reviewer #2: All is ok

**********

**Conclusions**

-Are the conclusions supported by the data presented?

-Are the limitations of analysis clearly described?

-Do the authors discuss how these data can be helpful to advance our understanding of the topic under study?

-Is public health relevance addressed?

Reviewer #1: It is not clear that methodology can be used as real forecasting.

It appears to be be more a model validation "Forecast of the past". See more in attached document.

Reviewer #2: See my report

**********

**Editorial and Data Presentation Modifications?**

Use this section for editorial suggestions as well as relatively minor modifications of existing data that would enhance clarity. If the only modifications needed are minor and/or editorial, you may wish to recommend “Minor Revision” or “Accept”.

Reviewer #1: Major revisions and editing.

See document.

Reviewer #2: Minor revisions

**********

**Summary and General Comments**

Use this section to provide overall comments, discuss strengths/weaknesses of the study, novelty, significance, general execution and scholarship. You may also include additional comments for the author, including concerns about dual publication, research ethics, or publication ethics. If requesting major revision, please articulate the new experiments that are needed.

Reviewer #1: See document attached.

Reviewer #2: Dear Authors (and editor),

tyour manuscript is very interesting but it has some problems.

So I request minor revisions

The key problem is the interpretation of figures 4 and 5

Indeed, you wrote that the Legend of figure 4 refers to the Hybrid model and that figure 5 refers to the Hybrid model.

Moreover you said that fig 5 performs much Better than fig 4

So you conclude that Hybrid model is Superior.

However, inspecting figure 4 and figure 5 I think that the best performance is related to figure 4 and not to figure 5

so, your must immediately clarify if you made errors In The Legends and in the text, or if you really think that the performance showed in figure five are the best one, which Would Surprise me a lot.

The above Is The Major problem of the ms

Other points:

The title seems to me not the best possible.

I suggest a New title which could be

"Advancing Outback detection for Dengue: hybridizing machine learning with wavelets for forecasting"

or something similar

ML and Wavelets must appear in the title!

In the abstract, the accuracy of the classical approach must Be rappresented as 74.2% and for the Hybrid model as 87.6%

Row 79: 595430

Row 148: cite the early papers on DWT, for example:

Dorize, C., Rioul, O., & Chaigne, A. (1989, August). Analysis/synthesis of sound signals using a discrete wavelet transform (DWT). In 13th International Congress on Acoustics.

Row 161: Instead of "recently" it is better "in last 20 years"

Row 114: there is an extra )

Row 201: add that youbtefere to Philippines

Figure 1: explain much better in the text, especially the rationale underlying the choice h=17

Row 225: add a reference for the python module "pmdarima"

Row 233: add a reference for Tensor flow

Row 233: did you also use Keras ? If yes, add a reference to the book by Chollet

Rows 277 to 279: use "Upper Panel:" and "Lower Panel"

Row 241: add a reference for Adam

Rows 244-245: the sentence " An experiment ..configuration" has no verb

**********

PLOS authors have the option to publish the peer review history of their article (what does this mean?). If published, this will include your full peer review and any attached files.

If you choose “no”, your identity will remain anonymous but your review may still be made public.

**Do you want your identity to be public for this peer review?**  For information about this choice, including consent withdrawal, please see our Privacy Policy.

Reviewer #1: No

Reviewer #2: No

[NOTE: If reviewer comments were submitted as an attachment file, they will be attached to this email and accessible via the submission site. Please log into your account, locate the manuscript record, and check for the action link "View Attachments". If this link does not appear, there are no attachment files.]

**Figure resubmission:**

While revising your submission, we strongly recommend that you use PLOS’s NAAS tool (https://ngplosjournals.pagemajik.ai/artanalysis) to test your figure files. NAAS can convert your figure files to the TIFF file type and meet basic requirements (such as print size, resolution), or provide you with a report on issues that do not meet our requirements and that NAAS cannot fix.

-->After uploading your figures to PLOS’s NAAS tool - https://ngplosjournals.pagemajik.ai/artanalysis, NAAS will process the files provided and display the results in the "Uploaded Files" section of the page as the processing is complete. If the uploaded figures meet our requirements (or NAAS is able to fix the files to meet our requirements), the figure will be marked as "fixed" above. If NAAS is unable to fix the files, a red "failed" label will appear above. When NAAS has confirmed that the figure files meet our requirements, please download the file via the download option, and include these NAAS processed figure files when submitting your revised manuscript.-->

**Reproducibility:**

To enhance the reproducibility of your results, we recommend that authors of applicable studies deposit laboratory protocols in protocols.io, where a protocol can be assigned its own identifier (DOI) such that it can be cited independently in the future. Additionally, PLOS ONE offers an option to publish peer-reviewed clinical study protocols. Read more information on sharing protocols at https://plos.org/protocols?utm_medium=editorial-email&utm_source=authorletters&utm_campaign=protocols

-->

Attachment Submitted filename: Review_PLOSNEG.pdf

# Author comment: Author response to Decision Letter 1

**DOI:** 10.1371/journal.pntd.0014444.r002

13 Nov 2025

Attachment Submitted filename: Response letter_1107FINAL.docx

# Peer review: Decision Letter 1

**DOI:** 10.1371/journal.pntd.0014444.r003

**Authors:** Benn Sartorius, Maira Aguiar, Sitara Ajjampur

5 Jun 2026

Dear Professor Nakamura,

We are pleased to inform you that your manuscript 'Advancing Outbreak Detection: Hybridizing Machine Learning with Wavelets for Weekly Dengue Case Forecasting' has been provisionally accepted for publication in PLOS Neglected Tropical Diseases.

Before your manuscript can be formally accepted you will need to complete some formatting changes, which you will receive in a follow up email. A member of our team will be in touch with a set of requests.

Please note that your manuscript will not be scheduled for publication until you have made the required changes, so a swift response is appreciated.

IMPORTANT: The editorial review process is now complete. PLOS will only permit corrections to spelling, formatting or significant scientific errors from this point onwards. Requests for major changes, or any which affect the scientific understanding of your work, will cause delays to the publication date of your manuscript.

Should you, your institution's press office or the journal office choose to press release your paper, you will automatically be opted out of early publication. We ask that you notify us now if you or your institution is planning to press release the article. All press must be co-ordinated with PLOS.

Thank you again for supporting Open Access publishing; we are looking forward to publishing your work in PLOS Neglected Tropical Diseases.

Best regards,

Sitara Swarna Rao Ajjampur

Section Editor

PLOS Neglected Tropical Diseases

Sitara Ajjampur

Section Editor

PLOS Neglected Tropical Diseases

Shaden Kamhawi

co-Editor-in-Chief

PLOS Neglected Tropical Diseases

orcid.org/0000-0003-4304-636XX

Paul Brindley

co-Editor-in-Chief

PLOS Neglected Tropical Diseases

orcid.org/0000-0003-1765-0002

***********************************************************

p.p1 {margin: 0.0px 0.0px 0.0px 0.0px; line-height: 16.0px; font: 14.0px Arial; color: #323333; -webkit-text-stroke: #323333}span.s1 {font-kerning: none

Reviewer's Responses to Questions

**Key Review Criteria Required for Acceptance?**

As you describe the new analyses required for acceptance, please consider the following:

**Methods**

-Are the objectives of the study clearly articulated with a clear testable hypothesis stated?

-Is the study design appropriate to address the stated objectives?

-Is the population clearly described and appropriate for the hypothesis being tested?

-Is the sample size sufficient to ensure adequate power to address the hypothesis being tested?

-Were correct statistical analysis used to support conclusions?

-Are there concerns about ethical or regulatory requirements being met?

Reviewer #2: I am happy with the revision

The answer to all questions on methods now is yes

Reviewer #3: The study, after the changes recommended by the previous reviewers achieves the goals as stated and adds to the general use of wavelets and machine learning.

One small consideration that the authors should consider either for the current submission or for future work is considering the question of foward looking bias. This is one of the many pitfalls in wavelet-based forecasting. The Daubechies-3 has support length 6, so each coefficient near the training boundary is computed using neighboring samples. If the authors decomposed the full series (train + test) once and then split — which is the most common implementation method — then some of the reported metrics may be optimistic. The text and Fig 1 say DWT was applied to the training set only, which is correct in principle, but the paper never explicitly states that the decomposition was redone in a rolling/expanding-window fashion as new data would arrive in real deployment. It would be more rigorous to explicitly demonstrate this, ideally with a walk-forward computation.

This is only a suggestion however and should not effect the publication.

**********

**Results**

-Does the analysis presented match the analysis plan?

-Are the results clearly and completely presented?

-Are the figures (Tables, Images) of sufficient quality for clarity?

Reviewer #2: I am happy with the revision

The answer to all questions on results now is yes

Reviewer #3: Figure 2, showing the wavelet coefficients could be made more useful if it displayed all coefficients, with perhaps the inclusion of a scalogram. This would allow readers to looks at results across all scales.

**********

**Conclusions**

-Are the conclusions supported by the data presented?

-Are the limitations of analysis clearly described?

-Do the authors discuss how these data can be helpful to advance our understanding of the topic under study?

-Is public health relevance addressed?

Reviewer #2: I am happy with the revision

The answer to all questions on comcludions now is yes

Reviewer #3: The public health relevance of the study is explicitly considered and the analysis is directed toward a very limited and specific goal. These two features certainly strengthen the paper and show the authors deep understabding of the central features of the prediction of Dengue transmission.

**********

**Editorial and Data Presentation Modifications?**

Use this section for editorial suggestions as well as relatively minor modifications of existing data that would enhance clarity. If the only modifications needed are minor and/or editorial, you may wish to recommend “Minor Revision” or “Accept”.

Reviewer #2: NA

Reviewer #3: One data consideration the authors might address in the future---but again not necessary for this publication. The next step could be to develop a deeper baseline comparison.Showing how and why DWT helps at all. A direct SARMA(-LSTM) on the raw weekly series is the obvious control. Without it, the wavelet step may be unjustified — most of the improvement from DWT-SARMA to DWT-SARMA-LSTM could simply be LSTM doing residual learning on any model. They could do: (i) SARMA-only, (ii) SARMA-LSTM (no wavelet), (iii) LSTM-only, alongside their two hybrids.

**********

**Summary and General Comments**

Use this section to provide overall comments, discuss strengths/weaknesses of the study, novelty, significance, general execution and scholarship. You may also include additional comments for the author, including concerns about dual publication, research ethics, or publication ethics. If requesting major revision, please articulate the new experiments that are needed.

Reviewer #2: All is now ok

Reviewer #3: The paper accomplishes it goals without methodological flaws and should be accepted. There are however places, outlined in my comments above where the methodology could be improved and more formalized as they move forward with this line research.

**********

PLOS authors have the option to publish the peer review history of their article (what does this mean?). If published, this will include your full peer review and any attached files.

If you choose “no”, your identity will remain anonymous but your review may still be made public.

**Do you want your identity to be public for this peer review?**  For information about this choice, including consent withdrawal, please see our Privacy Policy.

Reviewer #2: No

Reviewer #3: **Yes:**  John Hessler

# Editor report: Acceptance letter

**DOI:** 10.1371/journal.pntd.0014444.r004

**Authors:** Benn Sartorius, Maira Aguiar, Sitara Ajjampur

Dear Professor Nakamura,

We are delighted to inform you that your manuscript, "Advancing Outbreak Detection: Hybridizing Machine Learning with Wavelets for Weekly Dengue Case Forecasting," has been formally accepted for publication in PLOS Neglected Tropical Diseases.

We have now passed your article onto the PLOS Production Department who will complete the rest of the publication process. All authors will receive a confirmation email upon publication.

The corresponding author will soon be receiving a typeset proof for review, to ensure errors have not been introduced during production. Please review the PDF proof of your manuscript carefully, as this is the last chance to correct any scientific or type-setting errors. Please note that major changes, or those which affect the scientific understanding of the work, will likely cause delays to the publication date of your manuscript. Note: Proofs for Front Matter articles (Editorial, Viewpoint, Symposium, Review, etc...) are generated on a different schedule and may not be made available as quickly.

Soon after your final files are uploaded, the early version of your manuscript will be published online unless you opted out of this process. The date of the early version will be your article's publication date. The final article will be published to the same URL, and all versions of the paper will be accessible to readers.

For Research Articles, you will receive an invoice from PLOS for your publication fee after your manuscript has reached the completed accept phase. If you receive an email requesting payment before acceptance or for any other service, this may be a phishing scheme. Learn how to identify phishing emails and protect your accounts at https://explore.plos.org/phishing.

Thank you again for supporting open-access publishing; we are looking forward to publishing your work in PLOS Neglected Tropical Diseases.

Best regards,

Shaden Kamhawi

co-Editor-in-Chief

PLOS Neglected Tropical Diseases

Paul Brindley

co-Editor-in-Chief

PLOS Neglected Tropical Diseases
